# Tanshinone IIA Protects against Acute Pancreatitis in Mice by Inhibiting Oxidative Stress via the Nrf2/ROS Pathway

**DOI:** 10.1155/2020/5390482

**Published:** 2020-04-08

**Authors:** Weiwei Chen, Chenchen Yuan, Yingying Lu, Qingtian Zhu, Xiaojie Ma, Weiming Xiao, Weijuan Gong, Wei Huang, Qing Xia, Guotao Lu, Weiqin Li

**Affiliations:** ^1^Department of Gastroenterology, Clinical Medical College, Yangzhou University, Yangzhou, Jiangsu, China; ^2^Department of Critical Care Medicine, PLA Key Laboratory of Emergency and Critical Care Research, Jinling Hospital, Medical School of Nanjing University, Nanjing, Jiangsu, China; ^3^Department of Critical Care Medicine, PLA Key Laboratory of Emergency and Critical Care Research, Jinling Hospital, Medical School of Southeast University, Nanjing, Jiangsu, China; ^4^Pancreatic Center, Department of Gastroenterology, Affiliated Hospital of Yangzhou University, Yangzhou University, Yangzhou, Jiangsu, China; ^5^Department of Immunology, School of Medicine, Yangzhou University, Yangzhou, Jiangsu, China; ^6^Jiangsu Co-innovation Center for Prevention and Control of Important Animal Infectious Diseases and Zoonoses, College of Veterinary Medicine, Yangzhou University, Yangzhou, Jiangsu, China; ^7^Department and Laboratory of Integrated Traditional Chinese and Western Medicine, Sichuan Provincial Pancreatitis Centre and West China-Liverpool Biomedical Research Centre, West China Hospital, Sichuan University, Chengdu, China

## Abstract

**Background:**

Danshen (*Salvia miltiorrhiza* Bunge) and its main active component Tanshinone IIA (TSA) are clinically used in China. However, the effects of TSA on acute pancreatitis (AP) and its potential mechanism have not been investigated. In this study, our objective was to investigate the protective effects of TSA against AP via three classic mouse models.

**Methods:**

Mouse models of AP were established by caerulein, sodium taurocholate, and L-arginine, separately. Pancreatic and pulmonary histopathological characteristics and serum amylase and lipase levels were evaluated, and changes in oxidative stress injury and the ultrastructure of acinar cells were observed. The reactive oxygen species (ROS) inhibitor N-Acetylcysteine (NAC) and nuclear factor erythroid 2-related factor 2 (Nrf2) knockout mice were applied to clarify the protective mechanism of the drug.

**Results:**

In the caerulein-induced AP model, TSA administration reduced serum amylase and lipase levels and ameliorated the histopathological manifestations of AP in pancreatic tissue. Additionally, TSA appreciably decreased ROS release, protected the structures of mitochondria and the endoplasmic reticulum, and increased the protein expression of Nrf2 and heme oxygenase 1 of pancreatic tissue. In addition, the protective effects of TSA against AP were counteracted by blocking the oxidative stress (NAC administration and Nrf2 knockout in mice). Furthermore, we found that TSA protects pancreatic tissue from damage and pancreatitis-associated lung injury in two additional mouse models induced by sodium taurocholate and by L-arginine.

**Conclusion:**

Our data confirmed the protective effects of TSA against AP in mice by inhibiting oxidative stress via the Nrf2/ROS pathway.

## 1. Introduction

Acute pancreatitis (AP) is a common and aseptic inflammatory disorder of the pancreas. According to global estimates, the incidence of AP continues to rise, resulting in a substantial medical and social burden [1, 2]. In the majority of patients, AP is mild and self-limited; however, many patients suffered from pancreatic necrosis and multiple organ failure (mainly acute lung injury), which leads to a high mortality [3, 4]. Unfortunately, despite the great progress in drug research in recent years, there are almost no new drugs for the treatment of AP.

Traditional Chinese medicine is clinically used in the treatment of AP for a long history in China, such as Danshen (*Salvia miltiorrhiza* Bunge). The potential therapeutic effect on AP was shown in a small sample clinical trial [5]. As one of the main active components of *Salvia miltiorrhiza*, Tanshinone IIA (TSA) has been found effective in many diseases, such as cardiovascular diseases, liver diseases, and cancer [6–8]. TSA has been broadly studied for its multiple pharmacological properties including antiangiogenic, antioxidant, anti-inflammatory, and anticancer activities [7, 9, 10]. More importantly, its sulfonate sodium injection has been clinically applied in China for many years, especially in cardiovascular and cerebrovascular diseases. However, the effect of TSA against AP and its underlying mechanisms are unknown.

Excessive oxidative stress is one of the most important causes of cell injury in acute inflammatory diseases [11]. Studies have indicated that oxidative stress and the related production of reactive oxygen species (ROS) play a major role in pancreatic acinar cell injury in AP [12, 13]. Moreover, many studies suggest that antioxidant treatment can effectively reduce pancreatic tissue damage and inflammatory response in mice of AP, such as carbon monoxide-bound hemoglobin vesicle [14], coenzyme Q10 [15], and isoliquiritigenin we reported earlier [16]. However, it is far away from the clinical application.

In this study, we selected TSA, a traditional Chinese medicine monomer that has been used clinically, to investigate the protective effects on AP through three classic murine models and to explore the potential underlying mechanisms.

## 2. Materials and Methods

### 2.1. Animals

Male ICR mice weighing between 25 and 30 g were purchased from the Yangzhou University Model Animal Center (Yangzhou, China). C57BL/6 wild-type (WT) and nuclear factor erythroid 2-related factor 2 (Nrf2) knockout (KO) mice weighing between 20 and 25 g were purchased from the Model Animal Research Center of Nanjing University (Nanjing, China). Before the experiment, all mice were housed in specific pathogen-free (SPF) facilities, fed standard rodent chow and water, and maintained at a controlled temperature (25°C ± 2°C) under a light cycle (12 h light/12 h dark). This study was performed with the permission of the Science and Technology Commission of the Clinical Medical College of Yangzhou University and was carried out in accordance with the Principles of Laboratory Animal Care (NIH publication number 85-23, revised 1996).

### 2.2. Experimental AP Models and Drug Administration

In order to investigate the protective effects of TSA against AP, three classic mouse models of AP were established. All the three models in each independent experiment were repeated two times.

#### 2.2.1. Mild AP Model Induced by Caerulein

A mild AP (MAP) model was induced by intraperitoneal (i.p.) injection of caerulein (200 *μ*g/kg, 10 times at one-hour intervals; Cat. Pep03263, Nanjing Peptide Co. Ltd., Nanjing, China) [16, 17]. Mice were randomized into five groups: vehicle, caerulein, caerulein+low-dose TSA (5 mg/kg), caerulein+medium-dose TSA (25 mg/kg), and caerulein+high-dose TSA (50 mg/kg). TSA (Cat. HY-N1370-100 mg, MCE Co. Ltd., New Jersey, USA) dissolved in PBS was injected intraperitoneally 0.5 h before the first caerulein injection. An equal volume of PBS was injected into the vehicle and caerulein group mice. All groups of mice were sacrificed 12 h after the first injection of caerulein.

#### 2.2.2. Severe AP Model Induced by Sodium Taurocholate Hydrate (TLC)

In the TLC (Cat. T4009, Sigma-Aldrich, St. Louis, MO, USA) induced SAP model, retrograde infusion of TLC (1% TLC (100 *μ*l)+methylene blue reagent (20 *μ*l)) dissolved in saline was slowly injected into the distal common bile duct and pancreatic duct using an insulin needle [18]. All mice were randomized into three groups: sham, TLC, and TLC+TSA. In the sham group, only abdominal operation was performed, and TLC was not injected. In the TLC+TSA group, TSA (25 mg/kg) was injected intraperitoneally into mice 2 h before the operation. An equal volume of PBS was injected into the sham and TLC groups. All mice were sacrificed 12 h after TLC administration.

#### 2.2.3. Severe AP Model Induced by L-Arg (L-Arg)

In the SAP model induced by i.p. injection of L-Arg (4 g/kg, 2 times at a 1 h interval; Cat. A5006, Sigma-Aldrich, St. Louis, MO, USA) [16], mice were also randomized into three groups: vehicle, L-Arg, and L-Arg+TSA. After the first injection of L-Arg, a medium dose of TSA (25 mg/kg) was injected intraperitoneally at 24 h and 48 h. All mice were sacrificed 72 h after L-Arg administration.

### 2.3. Blocked the Oxidative Stress Pathway

To explore the involvement of TSA in mediating the Nrf2/ROS antioxidant pathway in AP in vivo, the ROS inhibitor N-Acetylcysteine (NAC) was administered to caerulein-induced mice, resulting in five groups: vehicle, caerulein, caerulein+TSA, caerulein+NAC, and caerulein+TSA+NAC. NAC (100 mg/kg, 2 times at a 1 h interval; Cat. No. A0737, Sigma-Aldrich, St. Louis, MO, USA) and TSA (25 mg/kg) dissolved in PBS were preadministered intraperitoneally 1 h and 0.5 h, respectively, before caerulein administration. Then, Nrf2 KO mice were incorporated into the caerulein-induced model, resulting in six groups: WT vehicle, WT caerulein, WT caerulein+TSA, KO vehicle, KO caerulein, and KO caerulein+TSA. TSA (25 mg/kg) was administered via the same method described above. All groups of mice were sacrificed 12 h after the first injection of caerulein.

### 2.4. Blood and Tissue Collection

All groups of mice were sacrificed after anesthetization via i.p. injection of sodium pentobarbital (50 mg/kg). Serum was obtained at different time points for amylase and lipase measurements according to the manufacturer's instructions (Amylase Kits (Cat. 100000060) were bought from Biosino Bio-Technology, Beijing, China; Lipase Kits (Cat. A054-1-1) were bought from Nanjing Jiancheng Corp., Nanjing, China). Pancreatic and pulmonary tissues were harvested immediately, some tissues were fixed in 4% paraformaldehyde for morphological study, and the other tissues were stored at −80°C for further analysis.

### 2.5. Histopathological Severity Evaluation

After dehydration, waxing, and embedding, pancreatic and pulmonary tissues were stained with hematoxylin and eosin, respectively. Histomorphological damage to the pancreas was assessed by the severity of edema, inflammatory cell infiltration, and acinar cell necrosis [19]. And histopathological damage to the lung was assessed by the severity of infiltration of neutrophils, thickness of alveolar, and alveolar congestion [20]. All the histopathological scoring evaluation was performed by two blinded pathologists.

### 2.6. Oxidative Stress Detection

In order to protect the oxidative stress indexes from oxidation, pancreatic tissues were homogenized in precooling PBS immediately after mice scarifying without multigelation and then centrifuged to obtain supernatant. All the procedures were operated on ice. Malondialdehyde (MDA) and glutathione (GSH) levels in the supernatant were measured according to the manufacturer's instructions (Cat. KGT004 and KGT006, Nanjing KeyGen Biotech Co. Ltd., Nanjing, China). The ROS generation in pancreatic tissues was detected by fluorescent probe-Dihydroethidium (DHE), and the detailed procedures were reported in our previous study [16].

### 2.7. Transmission Electron Microscopy

Furthermore, the ultrastructure of acinar cells, especially mitochondria and the endoplasmic reticulum, was observed by transmission electron microscopy. Small fresh pancreatic tissues (1 mm^3^) were instantly placed in an electron microscope fixative at 4°C for 2-4 h and then fixed in 1% citric acid·0.1 M phosphate buffer PB (pH 7.4) at 20°C for 2 h. After dehydration with alcohol and acetone, penetration with acetone and 812 embedding agents, and embedding and aggregation, the pancreatic tissues were cut into ultrathin slices (60-80 nm) by Leica UC7. Double staining of uranium-plumbum and dried overnight, the slices were observed under transmission electron microscopy.

### 2.8. Western Blotting

Referring to our previous methods [16], primary antibodies against Nrf2 (1 : 1000 dilution; Cat. ab31163, Abcam, Cambridge, UK), heme oxygenase (HO-1) (1 : 1000 dilution; Cat. ab68477, Abcam, Cambridge, UK), Lamin B1 (1 : 1000 dilution; Cat. 66095-I-IG, ProteinTech Group Inc., Chicago, USA), and *β*-actin (1 : 1000 dilution; Cat. sc47778, Santa Cruz, CA, USA) and secondary antibodies (1 : 5000 dilution; Cat. ab205719 and ab205718, Abcam, Cambridge, UK) were applied. Band densities were analyzed, and *β*-actin and Lamin B1 were used as loading controls.

### 2.9. Statistical Analysis

Statistical analysis was performed by a statistician blinded to this study with GraphPad Prism 6 software (GraphPad, San Diego, CA, USA), and data were presented as the mean ± SEM with vertical bars. The results were analyzed with one-way analysis of variance, Student-Newman-Keuls tests, and the Mann–Whitney rank sum test, and *p* < 0.05 was considered statistically significant.

## 3. Results

### 3.1. TSA Alleviated Pancreatic Histopathological Injury and Serum Enzymes in Caerulein-Induced AP

**Figure 1:**
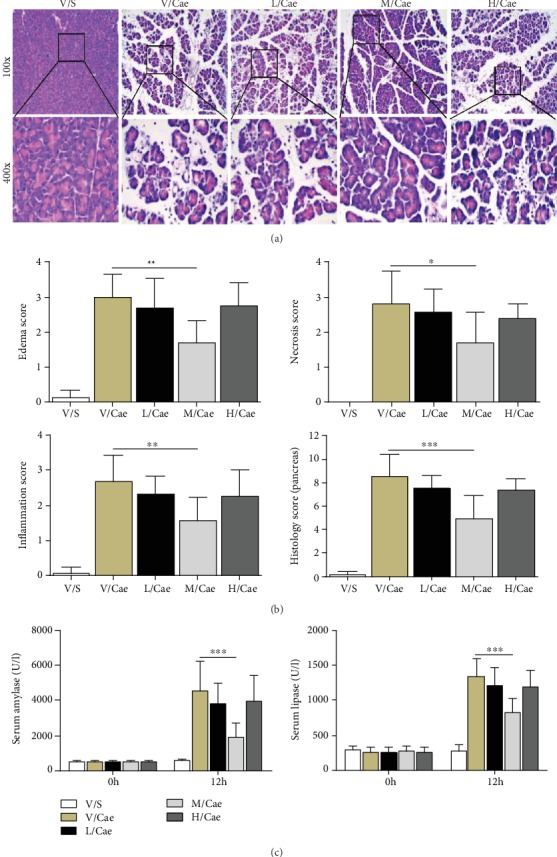
TSA alleviated pancreatic histopathological injury and serum enzymes in caerulein-induced AP. (a) Representative HE staining of pancreatic tissues in magnifications 100x and 400x. (b) Histopathological scores of pancreatic tissues. (c) Serum amylase and lipase levels. ^∗^*p* < 0.05, ^∗∗^*p* < 0.01, and ^∗∗∗^*p* < 0.001 versus the V/Cae group. *n* = 8 each group. V represents vehicle; S represents saline; L, M, and H represent low-dose (5 mg/kg), medium-dose (25 mg/kg), and high-dose (50 mg/kg) TSA.

As shown in Figures 1(a) and 1(b), TSA ameliorated the histological injury, resulting in decreased edema, inflammatory cell infiltration, and acinar cell necrosis. In addition, with a significantly statistic difference, medium-dose TSA (25 mg/kg) was more effective than either low-dose (5 mg/kg) or high-dose (50 mg/kg) TSA. These enzymatic results were consistent with the pancreatic histopathological severity assessment (Figure 1(c)). To verify the effect of TSA against AP and explore the underlying pathway, we selected medium-dose TSA for further experiments.

### 3.2. TSA Relieved the Oxidative Stress Injury and Mitochondrial Damage in Pancreatic Tissue in Caerulein-Induced AP

**Figure 2:**
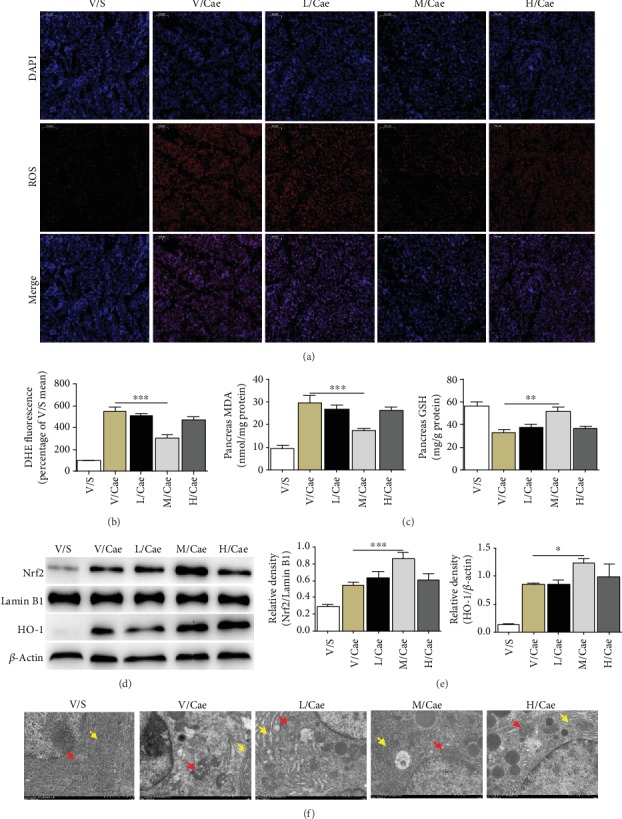
TSA relieved the oxidative stress injury and mitochondrial damage in pancreatic tissue in caerulein-induced AP. (a) Representative DHE immunofluorescence image of pancreatic tissues in magnification 100 *μ*m. (b) DHE fluorescence. Percentage of V/S mean. (c) Levels of oxidative stress products (MDA and GSH) of pancreatic tissues. (d, e) Protein levels of Nrf2 and total HO-1 in pancreatic tissues were analyzed by western blotting. (f) The organelles of pancreatic acinar cells via transmission electron microscopy. Red and yellow arrowheads indicate mitochondria and endoplasmic reticulum, respectively. ^∗^*p* < 0.05, ^∗∗^*p* < 0.01, and ^∗∗∗^*p* < 0.001 versus the V/Cae group. *n* = 8 each group. V represents vehicle; S represents saline; L, M, and H represent low-dose (5 mg/kg), medium-dose (25 mg/kg), and high-dose (50 mg/kg) TSA.

In order to clarify the mechanism of TSA, we examined the changes of oxidative stress pathway and related products in pancreatic tissue according to previous literature reports [21, 22]. Consistent with the pancreatic tissue histopathology, the ROS production was reduced after TSA treatment, with the most marked decrease in staining in the medium-dose TSA (25 mg/kg) group (Figures 2(a) and 2(b)). Meanwhile, we found that TSA increased the activation of Nrf2 and HO-1 with the changes of MDA and GSH (Figures 2(c)–2(e)). In addition, we observed the organelles of pancreatic acinar cells via transmission electron microscopy. Ultrastructural changes were observed in organelles such as mitochondria and the endoplasmic reticulum. Pancreatic acinar cells in the vehicle group exhibited normal acinar nucleoli, mitochondria, and rough endoplasmic reticulum. In the caerulein-induced AP group, marked mitochondrial rupture with loss of cristae and cystic expansion of the endoplasmic reticulum was observed. However, after medium-dose TSA treatment, the mitochondria and endoplasmic reticulum seemed to return to their normal form without swelling (Figure 2(f)). Taken together, these results indicated that TSA has a definite antioxidant effect on AP.

### 3.3. ROS Removal by NAC Abolished the Protective Effect of TSA against AP Induced by Caerulein

**Figure 3:**
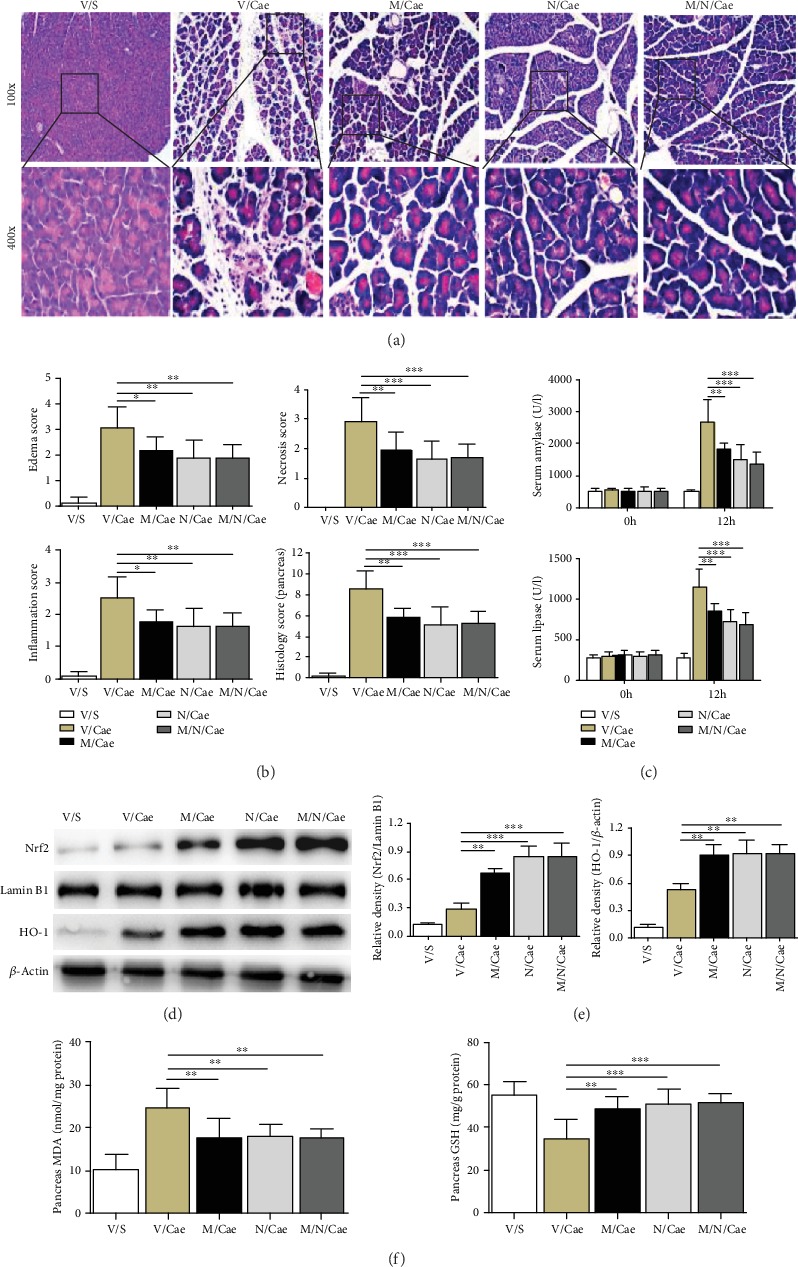
ROS removal by NAC abolished the protective effect of TSA against AP induced by caerulein. (a) Representative HE staining of pancreatic tissues in magnifications 100x and 400x. (b) Histopathological scores of pancreatic tissues. (c) Serum amylase and lipase levels. (d, e) Protein levels of Nrf2 and total HO-1 in pancreatic tissues were analyzed by western blotting. (f) Levels of oxidative stress products (MDA and GSH) of pancreatic tissues. ^∗^*p* < 0.05, ^∗∗^*p* < 0.01, and ^∗∗∗^*p* < 0.001 versus the V/Cae group. *n* = 8 each group. V represents vehicle; S represents saline; M represents medium-dose TSA (25 mg/kg); N represents ROS inhibitor NAC.

In order to further clarify the mechanism of drug action, the ROS production inhibitor NAC was applied. As shown in Figure 3, the pancreatic histopathological lesions, serum levels of enzymes (amylase and lipase), and level of MDA were significantly decreased after NAC treatment (Figures 3(a)–3(c) and 3(f)). In addition, the expressions of Nrf2 and HO-1 and the level of GSH were markedly higher after NAC administration (Figures 3(d)–3(f)). Obviously, on the basis of removing ROS by NAC, continued use of TSA did not show a protective effect on AP, nor did Nrf2/HO-1 protein expression level rise further. These results suggested that ROS regeneration plays a key role in the protection of TSA on AP.

### 3.4. The Protective Effect of TSA Was Counteracted in Nrf2 KO Mice in Caerulein-Induced AP

**Figure 4:**
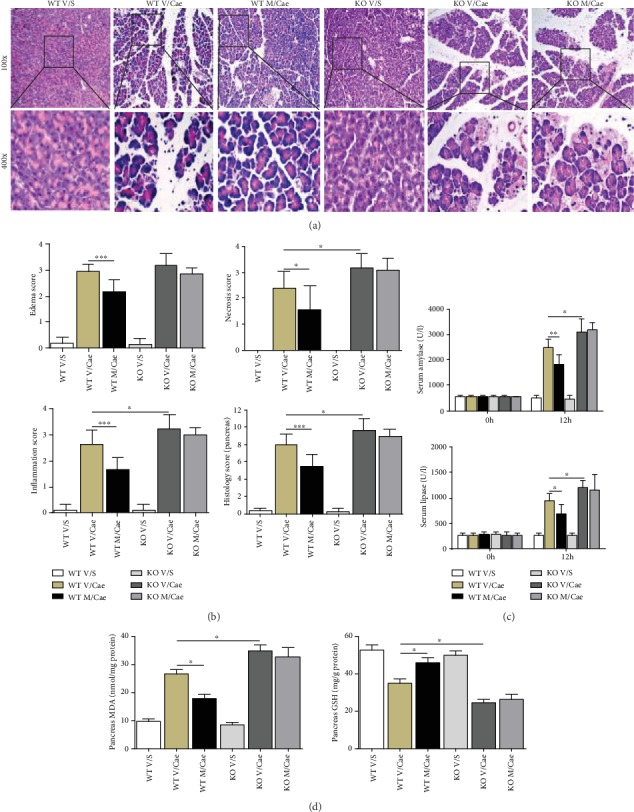
The protective effect of TSA was counteracted in Nrf2 KO mice in caerulein-induced AP. (a) Representative HE staining of pancreatic tissues in magnifications 100x and 400x. (b) Histopathological scores of pancreatic tissues. (c) Serum amylase and lipase levels. (d) Levels of oxidative stress products (MDA and GSH) of pancreatic tissues. ^∗^*p* < 0.05, ^∗∗^*p* < 0.01, and ^∗∗∗^*p* < 0.001 versus the V/Cae group. *n* = 8 each group. V represents vehicle; S represents saline; M represents medium-dose TSA (25 mg/kg).

Nrf2-deficient mice were used to further define the protective mechanism of TSA. As expected, pathological injury was more severe in Nrf2 KO mice than that in WT mice. After Nrf2 deficiency, the protective effects of TSA on pathological injury, enzymatic changes, and the levels of the oxidative stress products MDA and GSH were counteracted (Figures 4(a)–4(d)). Thus, with the above results of NAC treatment, TSA was confirmed to alleviate the severity of AP by activating the Nrf2/ROS pathway.

### 3.5. TSA Protected against SAP Induced by TLC and L-Arg

**Figure 5:**
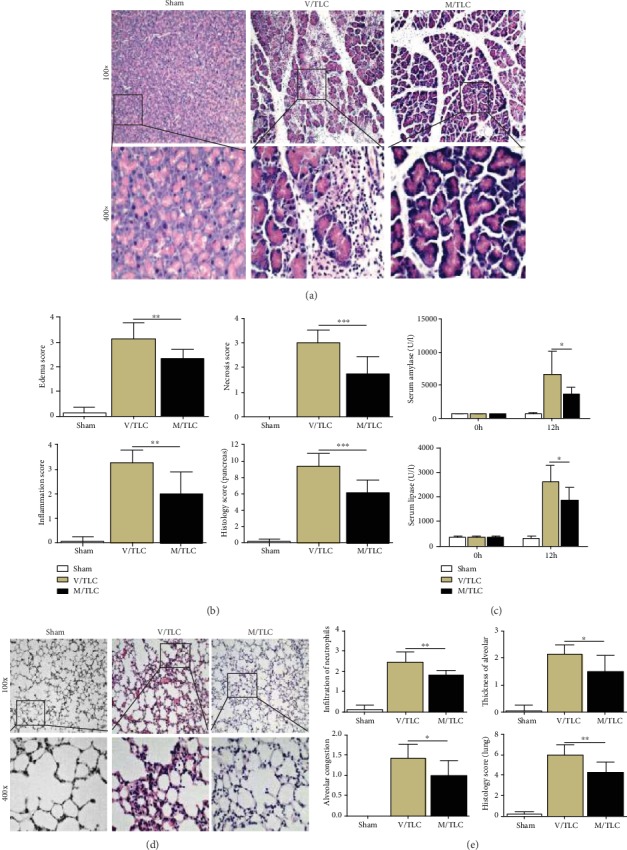
TSA protected against SAP induced by TLC. (a) Representative HE staining of pancreatic tissues in magnifications 100x and 400x. (b) Histopathological scores of pancreatic tissues. (c) Serum amylase and lipase levels. (d) Representative HE staining of pulmonary tissues in magnifications 100x and 400x. (e) Histopathological scores of pulmonary tissues. ^∗^*p* < 0.05, ^∗∗^*p* < 0.01, and ^∗∗∗^*p* < 0.001 versus the V/TLC group. *n* = 8 each group. V represents vehicle; M represents medium-dose TSA (25 mg/kg).

**Figure 6:**
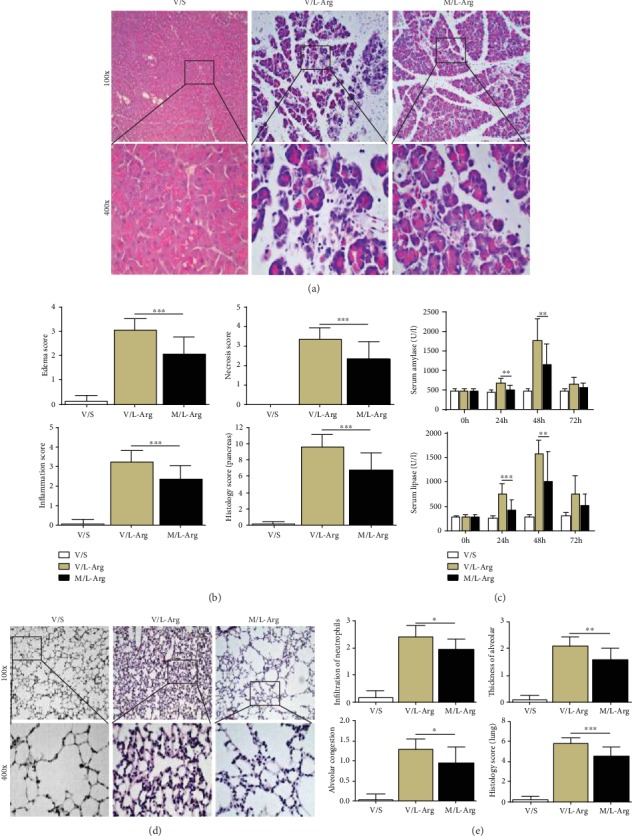
TSA protected against SAP induced by L-Arg. (a) Representative HE staining of pancreatic tissues in magnifications 100x and 400x. (b) Histopathological scores of pancreatic tissues. (c) Serum amylase and lipase levels. (d) Representative HE staining of pulmonary tissues in magnifications 100x and 400x. (e) Histopathological scores of pulmonary tissues. ^∗^*p* < 0.05, ^∗∗^*p* < 0.01, and ^∗∗∗^*p* < 0.001 versus the V/L-Arg group. *n* = 12 each group. V represents vehicle; S represents saline; M represents medium-dose TSA (25 mg/kg).

AP is an inflammatory reactive disease induced by many causes. In view of this, researchers often use a variety of different AP animal models to simulate the clinical reality of AP patients. Here, we choose two different AP models to verify the effectiveness of TSA, which will increase the feasibility of clinical application. Two mouse models of SAP were induced by TLC and L-Arg, separately. As expected, the severity of pancreatic tissue injury, including acinar cell necrosis, edema, and inflammatory cell infiltration, was obviously alleviated after TSA treatment in both TLC-induced and L-Arg-induced SAP models (Figures 5(a) and 5(b) and Figures 6(a) and 6(b)).

Meanwhile, the decrease of serum enzymology was observed (Figures 5(c) and 6(c)). Multiple organ failure represented by acute lung injury is one of the main causes of death in patients with AP. Therefore, lung histopathology was chosen to observe the severity of AP. It was found that SAP-associated acute lung injury was markedly reduced after TSA administration, characterized by less neutrophil infiltration, lower thickness of alveolar, and alleviated alveolar congestion (Figures 5(d), 5(e), 6(d), and 6(e)). Collectively, the above results indicated that TSA can protect mice against SAP in models induced by TLC and L-arginine.

## 4. Discussion

AP is a common acute abdominal disease of the digestive system with a high mortality rate for severe AP and lacks effective clinical treatment. So, it is urgent to develop effective drugs for the treatment of AP. It is a common method to find effective drugs from natural resources such as plants, animals, and microorganisms. Our previous study has reported that isoliquiritigenin can effectively reduce pancreatic tissue damage and inflammatory response in mice of AP [16]. However, it is not clinically used and far away from application. Hence, we focused on the Chinese herbs routinely used in clinical practice.

Danshen (*Salvia miltiorrhiza* Bunge), also known as red root, is one of the most commonly used Chinese herbals. It was first recorded in Shen Nong's herbal classic and has been used for thousands of years in China. In clinical practice, *Salvia miltiorrhiza* is a kind of traditional Chinese medicine which is often used to treat AP patients, and compound *Salvia miltiorrhiza* injection can decrease the expression of proinflammatory factors [23], improve the hemorheology abnormality, and reduce the acute respiratory distress syndrome and serious complications [5]. The protective effects of *Salvia miltiorrhiza* on pancreatic tissue damage and pancreatitis-related organ injuries were also observed in animal experiments [24]. However, the specific activity component of *Salvia miltiorrhiza* protecting against AP is still unknown. TSA is a liposoluble compound extracted from the root of *Salvia miltiorrhiza* and has many multiple biological activities. Its sulfonate sodium injection has been widely applied in clinical settings, mainly for treating cardiovascular and cerebrovascular diseases—for example, for improving vascular stiffness and blood pressure [25] and treating acute cerebral infarction and acute ischemic stroke [26, 27]. At present, there are few studies on the effect of TSA against AP. Our previous study showed that TSA could reduce the aortic endothelial damage in SAP rats [28]. Liu and Shen [29] found that TSA had a significant effect on SAP-related lung injury, which may be related to the change of cytokine level and the reduction of inflammatory cell infiltration in the lung. However, the effect of TSA on pancreatic necrosis and related underlining mechanism is still not clear.

In this study, our results demonstrated that TSA exerts protective effects in three classic AP models induced by caerulein, TLC, and L-Arg. We confirmed that prophylactic injection of TSA can ameliorate pancreatic pathological injury, serum enzymatic responses, and related acute lung injury. These results clearly showed the therapeutic effects of TSA against AP.

Although the pathogenesis of AP is fully unclear, Nrf2/HO-1-mediated oxidative stress and ROS generation are considered an underlying mechanism of AP [30–33]. Our previous study indicated that oxidative stress and Nrf2/HO-1 pathway were dynamically changed in pancreatic tissue of AP mice and were the most significant at the peak of inflammatory response [16]. Many clinical studies and animal experiments also showed the role of oxidative stress and ROS generation in the pathophysiological response of AP, activation of Nrf2/HO-1 pathway, and reduction of ROS generation, which showed the protective effect against AP [33–36]. Additionally, after reanalyzing the sequencing results of relevant research [37], we found that HO-1 increased significantly in the AP mouse model induced by caerulein (3.38-fold change *vs.* normal, *p* = 0.0001), while other related genes including Keap1, NQO1, and GCLM had no significant changes. Accordingly, the HO-1 gene was addressed as the Nrf2 target gene. In this study, the results showed that the Nrf2/HO-1 pathway was activated with the changes of ROS production and oxidative stress products in pancreatic tissue of AP, consistent with the results of our previous study [16]. TSA administration upregulated the expression of the Nrf2/HO-1 pathway, reduced the production of ROS, and protected the mitochondrial damage. All these results showed that TSA had a definite effect on reducing oxidative stress.

Our previous results showed that ML385, an Nrf2 inhibitor, can inhibit the activity of Nrf2. Nevertheless, what is puzzling is that ML385 protects the severity of pancreatitis in mice [16]. We surmised that ML385 may protect against AP through other nonproven pathways, which suggests that ML385 may not be a good choice for studying Nrf2 in the study of AP. Hence, in this present study, we used Nrf2-deficient mice and the ROS scavenger NAC, which is more reliable than ML385 and better for the verification of the mechanism of drug action. The results showed that removal of ROS generation or Nrf2 defects could counteract the protective effect of TSA. Accordantly, the mechanism of TSA was preliminarily defined.

**Figure 7:**
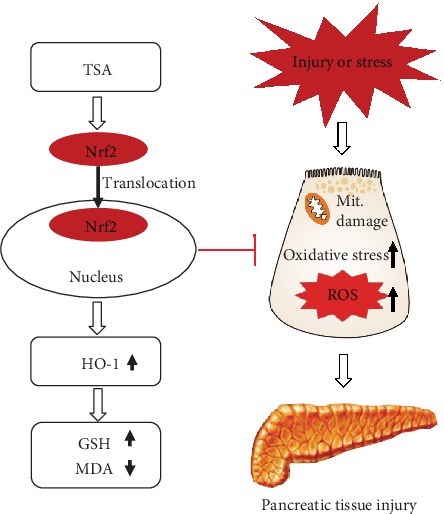
The potential Nrf2/ROS pathway of TSA protects against acute pancreatitis.

In conclusion, our findings first demonstrated that TSA effectively protects against AP by inhibiting oxidative stress via the Nrf2/ROS pathway (Figure 7). These results suggest that TSA is a promising therapeutic drug for AP in future clinical practice especially in the situation of TSA sulfonate sodium injection which has been applied clinically.

## Data Availability

All data used to support the findings of this study are available from the corresponding author upon request.
